# Preliminary Interpretation of the Induction Period in Hydration of Sodium Hydroxide/Silicate Activated Slag

**DOI:** 10.3390/ma13214796

**Published:** 2020-10-27

**Authors:** Yibing Zuo, Guang Ye

**Affiliations:** 1 School of Civil and Hydraulic Engineering, Huazhong University of Science and Technology, Wuhan 430074, China; 2 Faculty of Civil Engineering and Geosciences, Delft University of Technology, Stevinweg 1, 2628 CN Delft, The Netherlands; g.ye@tudelft.nl

**Keywords:** induction period, alkali-activated slag, soluble Si, dissolution, isothermal calorimetry

## Abstract

Many calorimetric studies have been carried out to investigate the reaction process of alkali-activated slag paste. However, the origin of the induction period and action mechanism of soluble Si in the dissolution of slag are still not clear. Moreover, the mechanisms behind different reaction periods are not well described. In this study, the reaction kinetics of alkali-activated slag paste was monitored by isothermal calorimetry and the effect of soluble Si was investigated through a dissolution test. The results showed that occurrence of the induction period in hydration of alkali-activated slag paste depended on the presence of soluble Si in alkaline activator and the soluble Si slowed down the dissolution of slag. A dissolution theory-based mechanism was introduced and applied to the dissolution of slag, showing good interpretation of the action mechanism of soluble Si. With this dissolution theory-based mechanism, origin of the induction period in hydration of alkali-activated slag was explicitly interpreted.

## 1. Introduction

The technology of alkali activation of ground granulated blast furnace slag (GGBFS) has been increasingly used for producing a clinker-free cementitious material, since it is able to efficiently improve the sustainability of concrete production [1,2,3,4]. Awoyera and Adesina reviewed the application of alkali-activated slag in the view point of its sustainability and summarized that alkali-activated slag cement contributes to achieve a greener concrete, taking off the strain on exploration of natural resources for ordinary Portland cement (OPC) production and preventing not only environmental degradation due to the exploration but also contamination due to the disposal of slag [5]. The alkali-activated slag cement has the ability to address the energy and environmental concerns that are associated with OPC production. It is reported that 80% or greater reduction of CO_2_ emission can be achieved by alkali-activated slag cement through proper use of alkaline activator when compared to OPC [6]. Yang et al. assessed the CO_2_ reduction of alkali-activated slag concrete and found 55–75% reduction of CO_2_ emission relative to OPC concrete [7]. The CO_2_ reduction is mainly because of the absence of a high-temperature calcination step in the production of alkali-activated cement. Compared with OPC, alkali-activated slag cement exhibits similar or even better mechanical properties and durability performance [8,9,10]. All of these excellent properties are closely related to the reaction process of alkali-activated slag, because the reaction process governs the reaction products formation, microstructure development and thus microstructure-related-physical-properties, including mechanical properties and durability performance [11,12,13].

Isothermal calorimetry, as a well-accepted technique for studying the reaction process, has been successfully and prevalently applied in cementitious systems. Many isothermal calorimetric studies have been conducted to investigate the hydration process of OPC, such as the influence of moisture [14] and temperature [15], incorporation of cement replacement materials [16] as well as chemical admixtures [17], etc. As a latent hydraulic cementitious material, GGBFS is widely used to replace OPC in concrete production. In addition to the calorimetric responses that reflect the hydration of OPC, the blended systems of OPC and slag also show a calorimetric peak that reflects the reaction of slag [18,19]. The similar calorimetric responses, reflecting reaction of slag, are expected in alkali-activated slag cement. Compared with those of OPC based systems, the calorimetric responses of alkali-activated slag cement could be very different, particularly depending on the nature and concentration of alkaline activators.

By studying the reaction kinetics of *powder* sodium silicate activated slag pastes and *liquid* sodium silicate activated slag pastes, the researchers found that both the *powder* sodium silicate activated slag pastes and the NaOH activated slag pastes showed a single calorimetric peak [20]. The *liquid* sodium silicate activated slag pastes and OPC pastes, however, both demonstrated multiple calorimetric peaks and an obvious induction period. In this study the induction period is defined as the duration between two calorimetric peaks on heat evolution curve, in which the rate of heat evolution is very low. The different calorimetric responses between *powder* and *liquid* sodium silicate activated slag pastes is resulted from a larger pH in the *powder* sodium silicate activator than that in the *liquid* sodium silicate activator (henceforth if not specified *liquid* sodium silicate is written as sodium silicate for clarity). In another study, the in-situ isothermal calorimetry was used to monitor the reaction kinetics in sodium hydroxide and sodium silicate activated slag pastes [21]. The in-situ data demonstrated only one major calorimetric peak with no induction period for sodium hydroxide activated slag pastes and multiple calorimetric peaks with an obvious induction period for sodium silicate activated pastes. Shi and Day [22] used a conduction calorimeter to monitor the reaction kinetics of alkali-activated slag pastes with different kinds of alkaline activators, such as NaOH, Na_2_CO_3_, Na_2_SiO_3_·5H_2_O, Na_3_PO_4_ and NaF. The calorimetric responses were dependent on the type of alkaline activator, as described by three proposed models by the researchers.

For the reaction kinetics of alkali-activated blends of slag with other aluminosilicate precursors, Gao et al. studied the sodium silicate activated slag-fly ash paste [23] and Bernal et al. investigated the sodium silicate activated slag-metakaolin paste [24]. It can be found that in the viewpoint of the trend of heat evolution rate curve, both the sodium silicate activated slag-fly ash pastes and the sodium silicate activated slag-metakaolin pastes showed similar calorimetric signals to those of sodium silicate activated slag paste, as long as slag was the major component in the blends.

**Figure 1:**
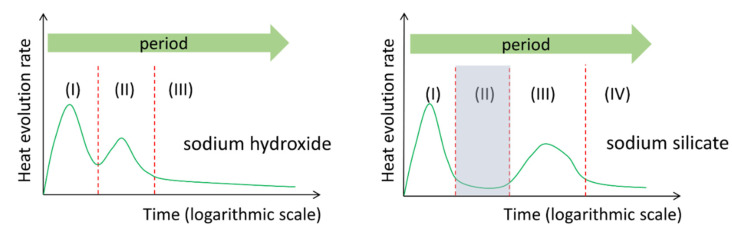
Schematic representation of the heat evolution rate curves of NaOH activated slag paste and sodium silicate activated slag paste. The shadowed part refers to the induction period.

According to the aforementioned calorimetric studies, among others in the literature like [25,26,27,28], an obvious different calorimetric characteristic can be found between the NaOH activated slag paste and the sodium silicate activated slag paste. As shown in Figure 1, the heat evolution rate curve of NaOH activated slag paste demonstrates no induction period, while that of sodium silicate activated slag paste show an obvious induction period. This difference can be attributed to the presence of soluble Si in sodium silicate activator. However, the action mechanism that how soluble Si results in an induction period is still not clear. Furthermore, the mechanisms behind different reaction periods are not well described and understood.

Therefore, this study aims to discuss and explicitly interpret the origin of the induction period and describe the mechanisms behind different reaction periods of alkali-activated slag paste. First, isothermal calorimetric experiments were carried out to monitor the calorimetric responses of sodium hydroxide activated slag pastes and sodium silicate activated slag pastes. Then, the effect of soluble Si on the dissolution of slag in alkaline solution was investigated through a dissolution test. In the dissolution test a recipe was carefully designed via thermodynamic analysis to avoid formation of any reaction products during the dissolution of slag. Afterwards a dissolution theory-based mechanism was introduced and applied to the dissolution of slag. Finally, the reaction process and origin of the induction period were described and interpreted, respectively. The results presented in this study will contribute to understanding the reaction kinetics of alkali-activated slag paste, particularly the effect of alkaline activator composition on the dissolution of slag.

## 2. Materials and Methods

### 2.1. Materials and Mixtures

**Figure 2:**
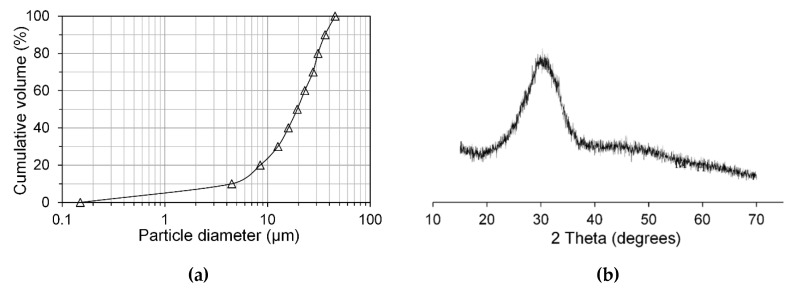
Particle size distribution (**a**) and X-ray diffraction pattern (**b**) of slag, determined by laser-diffraction and diffractometer, respectively.

In this study, GGBFS, from ORCEM The Netherlands, was used to prepare alkali-activated slag pastes. Table 1 presents the chemical composition of slag, as determined by X-ray fluorescence spectrometry (XRF) through Epsilon 3XLE spectrometer (PANalytical, Heracles Almelo, The Netherlands) test. The surface area was determined as 2.38 m^2^/g by Gemini VII 2390 (micromeritics, Brussel, Belgium). Figure 2 depicts the particle size distribution and X-ray diffraction pattern of slag, measured by EyeTech laser-diffraction (Ankersmid, Nijverdal, The Netherlands) and PW 1830X-ray diffractometer (Philips, Ameterdam, The Netherlands), respectively. It is clear that no crystalline phase was identified from the X-ray pattern, indicating a totally amorphous nature of slag. This result is in line with the fact that usually GGBFS contains >95% vitreous phase [29].

Sodium hydroxide (analytical grade, >98%, from Sigma-Aldrich, Darmstadt, German) was mixed with distilled water to prepare sodium hydroxide activator. Water glass (8.25 wt % Na_2_O, 27.5 wt % SiO_2_ and 64.25 wt % H_2_O, from BRENNTAG, Apolda, Belgium), combined with sodium hydroxide and distilled water, was used to prepare sodium silicate activator with the desired contents of Na_2_O and SiO_2_. According to the presence of soluble Si, the alkaline activators are divided into two groups. One group is sodium hydroxide activator without soluble Si and the other group is sodium silicate activator with soluble Si. Table 2 presents the mix compositions of the alkali-activated slag pastes. According to the contents of Na_2_O and SiO_2_, the mixtures were denoted as N4S0, N4S5.4, N6S0, N6S5.4, N8S0 and N8S5.4. The numbers following N and S indicate mass percentages of Na_2_O and SiO_2_ in the alkaline activator with respect to slag, respectively. For instance, sample N4S5.4 represents the alkali-activated slag paste with Na_2_O/slag = 4% and SiO_2_/slag = 5.4%. For all samples the water/slag ratio was fixed at 0.4.

### 2.2. Isothermal Calorimtry Test

By following the procedures in ASTM C1679 [30], the heat release flow of alkali-activated slag pastes was measured by an isothermal calorimeter. Alkaline activator solutions were formulated and cooled down to the temperature of 20 °C prior to mixing with slag. The alkali-activated slag pastes were prepared by mixing slag and alkaline activator solution externally and about 10 g of fresh paste was immediately put into the isothermal calorimeter (TAM-Air-314). The measurement temperature was set at 20 °C. For each mixture, two samples from the same mixing batch were tested at the same time. The duration of measurement was one week.

### 2.3. Dissolution Test

The dissolution test was carried out to study the effect of soluble Si on the dissolution of slag in alkaline solution. In the test, 0.1 g of slag was dissolved in 200 mL of alkaline solution. The temperature was fixed at 20 °C and a magnetic stirring of 250 rpm was applied to prevent particle aggregation. Three types of alkaline solution were used. The first type is 0.1 mol/L sodium hydroxide solution without soluble Si. The second and third types are 0.1 mol/L sodium hydroxide solutions with soluble Si by the concentrations of 1 mmol/L and 5 mmol/L, respectively. During the dissolution process, a small volume of solution was sampled with a syringe at different times. In order to remove the undissolved slag, filter paper was used to filter the sampled solution. Prior to element analysis in solution, the filtered solutions were diluted using nitric acid (2.0 vol.%). Afterwards, the diluted solutions were analyzed using a PerkinElmer Optima 5300DV ICP-OES spectrometer, based on which the concentrations of Si, Al and Ca in solution were obtained.

### 2.4. Scanning Electron Microscopy (SEM)

For SEM sample preparation, the samples were crushed into small pieces (1–2 cm^3^), then impregnated using a low viscosity epoxy resin and afterwards polished down to 0.25 μm (for more information on preparing SEM samples, please refer to [31]). Then, the microstructure of the polished samples was examined by ESEM (Philips XL30, Eindhoven, The Netherlands). The obtained SEM images with a magnification of 500× were used to determine the degree of reaction of slag through image analysis. More details on the image analysis method are provided in [32]. Moreover, the SEM images with a magnification of 1000× were also recorded for more clearly observing the microstructure morphology of alkali-activated slag pastes.

### 2.5. Thermodynamic Analysis of Solution

Thermodynamic analysis of solution was conducted to prove that no reaction products precipitated during the dissolution test in Section 2.3. In thermodynamic analysis, the saturation index (*SI*) was employed as a criteria to judge if reaction products can precipitate or not [33]:

*SI* = log(*IAP*/*K_S0_*) (1)

where *IAP* and *K_S0_* are the ion activity product and equilibrium solubility product of a solid phase, respectively. If *SI* > 0 it means this solid phase is likely to precipitate. If *SI* < 0, it indicates that this solid phase is not likely to precipitate. If *SI* = 0, it implies equilibrium between solution and solid. It should be noted that *SI* may result in confusions if someone wants to use it to analyze the precipitation probabilities of phases that dissociate into a different number of ions (*N*) [33]. Therefore, effective saturation index (ESI) was introduced:

*ESI* = *SI*/*N* (2)

In order to calculate the activities of ions, the Gibbs energy minimization software GEM-Selektor v.3 (http://gems.web.psi.ch/) [34,35] and the thermodynamic database in [36,37] for alkali-activated slag systems were used. The measured elemental concentrations were used as the input. Given the activities of ions, the ion activity products can be calculated according to the dissociation reactions of reaction products. With the ion activity products and equilibrium solubility products, the effective saturation index can be determined via Equation (2).

#### The Considered Reaction Products in the Thermodynamic Analysis

In alkali-activated slag cement, the reaction products can be categorized into primary reaction products and secondary reaction products. The primary reaction product is a calcium-sodium aluminosilicate hydrate (CNASH) [38]. Myers et al. used eight CNASH solid solution (CNASH_ss) end-members to model this primary reaction product [36]. These eight CNASH_ss end-members and their dissociation reactions and equilibrium solubility products are given in Table 3.

The secondary reaction products are crystalline phases that are identified in alkali-activated slag cement, such as hydrotalcite [40], tetracalcium aluminate hydrate (C_4_AH_13_) [40], katoite (C_3_AH_6_) [37], stratlingite (C_2_ASH_8_) [41] and portlandite (CH) [42]. The dissociation reactions and equilibrium solubility products of these crystalline phases except hydrotalcite are also given in Table 3.

## 3. Results

### 3.1. Reaction Kinetics of Alkali-Activated Slag Paste

**Figure 3:**
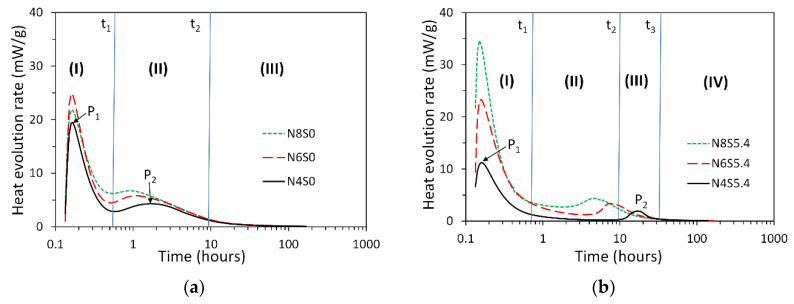
Heat evolution rates for sodium hydroxide (**a**) and sodium silicate (**b**) activated slag pastes. In the graph, t_1_, t_2_ and t_3_ stand for the transition time.

The measured heat evolution rates for sodium hydroxide and sodium silicate activated slag pastes are plotted in Figure 3a,b, respectively. For NaOH activated slag pastes (Figure 3a), the heat release rate curves demonstrate two calorimetric peaks, between which there is no noticeable induction period. These observations are in line with [12,20,21,22,43]. The first calorimetric peak, i.e., P_1_, occurred in the first ten minutes. It results from the dissolution/wetting of slag in sodium hydroxide solution [20,43]. The second calorimetric peak, i.e., P_2_, appeared around 1~3 h after mixing. According to the literature [22,43], P_2_ results from the formation of a large amount of reaction products. An increase of Na_2_O content in the sample led to a higher P_2_. Based on P_1_ and P_2_, three reaction periods can be identified during the reaction process of sodium hydroxide activated slag paste. As shown in Figure 3a, these three reaction periods are the initial dissolution period (I), acceleration/deceleration period (II) and steady period (III), respectively.

For sodium silicate activated slag paste (Figure 3b), an obvious induction period appeared between the two calorimetric peaks. This is obviously different from the calorimetric response for sodium hydroxide activated slag pastes. An increase of Na_2_O content in the sample led to both a higher P_2_ and earlier occurrence of P_2_. According to P_1_ and P_2_, the reaction process of sodium silicate activated slag paste had four reaction periods, i.e., the initial dissolution period (I), induction period (II), acceleration/deceleration period (III) and steady period (Ⅳ), respectively.

### 3.2. Dissolution Results

Table 4 lists the measured concentrations of Si, Al and Ca for the dissolution of slag in 0.1 mol/L sodium hydroxide solution with and without soluble Si. The thermodynamic analysis of solution will be carried out and the effect of soluble Si on the dissolution of slag will be discussed in the next two sections, respectively.

#### 3.2.1. Dissolution Results

**Figure 4:**
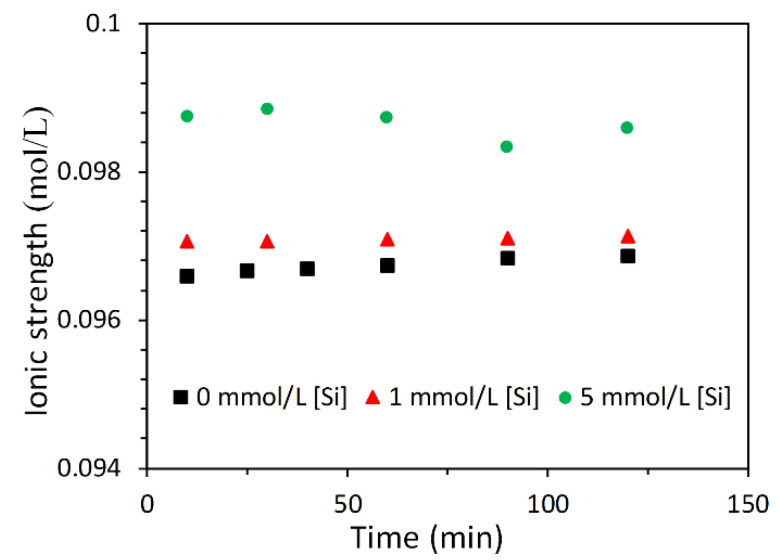
Ionic strength of solution.

The equilibrium solubility products of the eight CNASH_ss end-members and secondary reaction products in Table 3 were used to calculate the ESI as described in Section 2.5. Since the concentration of Mg in solution was below the detection limit and thus not measured, the ESI with respect to hydrotalcite was not calculated. It is noted that the ionic strength of solution was below 1.0 mol/L (see Figure 4), which is within the valid range (~1–2 mol/L) of the activity correction using the extended Debye–Huckel equation [44].

**Figure 5:**
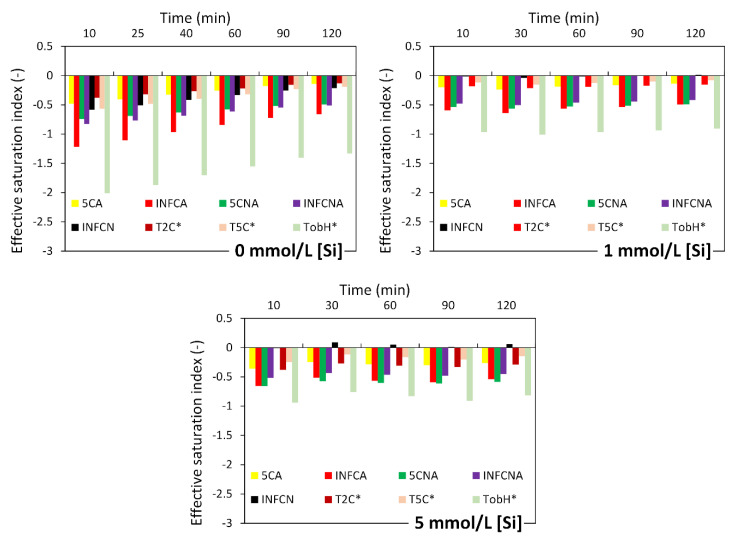
Calculated effective saturation indexes (ESI) for primary reaction products for the dissolution of slag in 0.1 mol/L sodium hydroxide solution with and without soluble Si.

**Figure 6:**
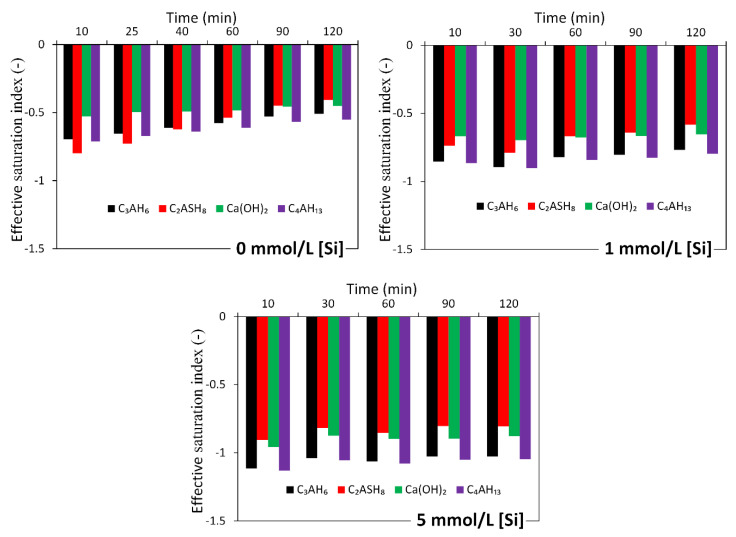
Calculated effective saturation indexes (ESI) for secondary reaction products for the dissolution of slag in 0.1 mol/L sodium hydroxide solution with and without soluble Si.

Figure 5 and Figure 6 present the calculated effective saturation indexes (*ESI*). Most of the ESI were smaller than 0. This means that the solutions were undersaturated with respect to all considered reaction products in alkali-activated slag cement. Therefore, these reaction products are unstable in the solutions and thus are not expected to precipitate. In other words, the elements like Si, Al and Ca, etc. were in the solution after dissolution from slag.

**Figure 7:**
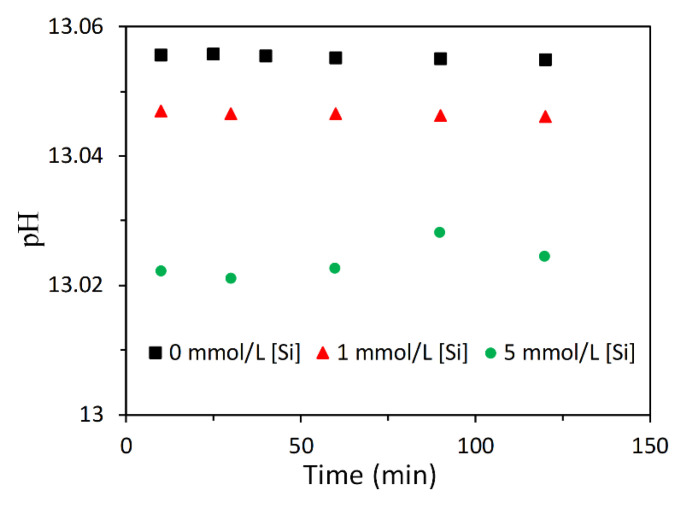
Calculated pH.

The calculated pH of solutions is presented in Figure 7. Although the addition of Si in sodium hydroxide solution decreased the pH, the reduction was smaller than 0.04. This tiny decrease of pH is not supposed to significantly affect the dissolution rate of slag. In other words, the dissolution of slag can only be influenced by the presence of soluble Si. As discussed previously that no reaction products precipitated and the elements like Si, Al and Ca, z. were all in the solution after dissolution, then variations of the concentrations of Ca and Al are able to reflect the dissolution degree of slag. Larger concentrations of Ca and Al means a larger dissolution degree of slag. Based on the variations of the concentrations of Ca and Al, the effect of soluble Si on the dissolution of slag can be deduced. This aspect will be discussed in details in the next subsection.

#### 3.2.2. Effect of Soluble Si on the Dissolution of Slag

**Figure 8:**
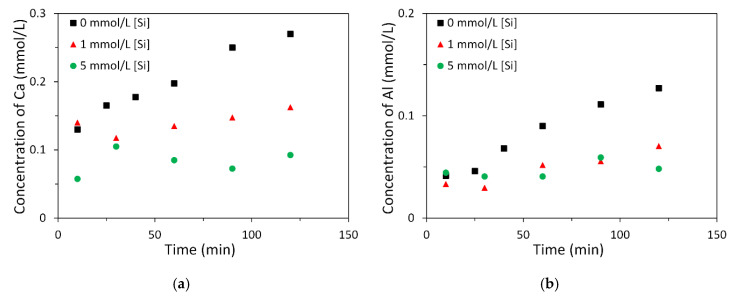
Measured concentrations of Ca (**a**) and Al (**b**) for the dissolution of slag in 0.1 mol/L sodium hydroxide solution with and without soluble Si.

Figure 8 displays the measured concentrations of Ca and Al for the dissolution of slag in 0.1 mol/L sodium hydroxide solution with and without soluble Si. It can be seen that the concentration of Ca was reduced when more soluble Si was added into the sodium hydroxide solution. The added soluble Si in the sodium hydroxide solution was also found to result in a decrease of the concentration of Al. Since all the dissolved elements were in the solution, reductions of the concentrations of Ca and Al reflect the retarded dissolution of slag due to the soluble Si. In other words, the soluble Si in alkaline solution slowed down the dissolution of slag.

The retarding effect of soluble Si on dissolution of slag then slowed down the reaction kinetics of sodium silicate activated slag, resulting in an induction period during the reaction process as shown in Figure 3b. The induction period has been also widely detected through isothermal calorimetry measurements by many studies in the literature [20,21,22,25,26,27,28]. Besides the reaction kinetics, the effect of soluble Si by slowing down the dissolution of slag also affects the microstructure formation. Many studies reported a homogenous microstructure in sodium silicate activated slag as opposed to a heterogeneous microstructure in sodium hydroxide activated slag [32,45,46]. One reason is that the slowing down dissolution of slag and thus retarded reaction kinetics leaves ample time for reaction products to form evenly in sodium silicate activated slag. Another reason is that the soluble Si can act as nucleation sites.

## 4. Discussion

### 4.1. Dissolution of Slag

**Figure 9:**
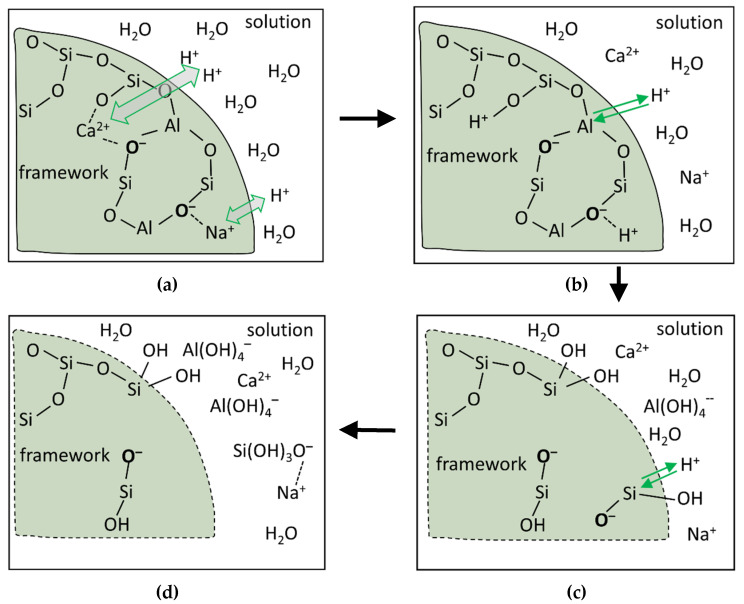
Schematic illustration of the dissolution of slag (after [51]). For clarity, additional bonds between Si and O as well as between Al and O are not shown. First, the modifying elements are initially released through the metal/proton exchange reactions, as shown in (**a**); then, hydrolysis of the bonds between Al and O starts, as shown in (**b**); afterwards, the bonds between Si and O start to break, as shown in (**c**); finally, Al and Si are released, as a result of which the framework is gradually dissolved, as shown in (**d**).

In aluminosilicate materials including slag, Si and Al build up the framework while alkali and alkali-earth metals like Ca and Mg modify the framework [47,48]. So, the alkali and alkali-earth metals are also called modifying elements. The framework refers to the glass network in aluminosilicate materials. In the framework Si and Al are tetrahedrally coordinated. As schematically shown in Figure 9, the dissolution of slag can be described via the following four steps [49,50,51].

Due to the smaller bonding energy of Al-O than Si-O, Al dissolves more easily than Si in the dissolution of slag [47]. The initially dissolved Al changes the adjoined Si coordination condition from fully coordinated to partially coordinated (Figure 9c). Compared with the fully coordinated Si, the partially coordinated Si dissolves faster. So the dissolution of framework can be divided into the following two steps: initial release of a small amount of Al (Figure 9b) and then followed by the release of Si that coordinates to the initially dissolved Al through O (Figure 9c).

**Figure 10:**
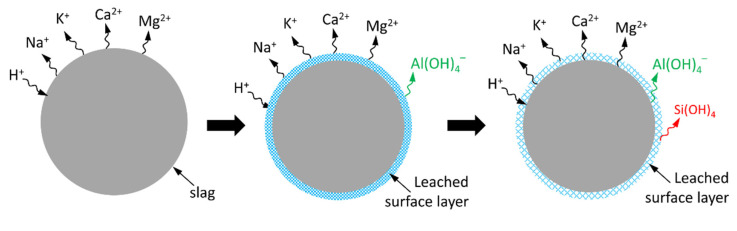
Schematic representation of the formation of the leached surface layer.

Due to the preferential dissolution of modifying elements, a leached surface layer is formed around the particle [47,48], as shown in Figure 10. This leached surface layer is mostly composed of leftover Al and Si and it affects the dissolution of slag since it influences how fast the modifying elements diffuse through it. A larger thickness of the leached surface layer obviously led to a smaller diffusion rate of the modifying element.

### 4.2. A Dissolution Theory Based Mechanism Applied to the Dissolution of Slag

Based on the theory of Lasaga and Luttge [52] and the “vacancy island” theory of Dove et al. [53], Juilland et al. put forward a dissolution theory based mechanism to explain the onset of the induction period in hydration of crystalline alite [54]. In this mechanism the dissolution is divided into three forms, i.e., the formation of vacancy islands, etch pit formation at dislocations and step retreat at pre-existing roughness. Crystallographic defects (acting as dislocations) and solution saturation are two main factors considered in this mechanism. Although there is no crystallographic defects in slag because of its amorphous nature, preferential dissolution of modifying elements and partially dissolved framework (see Figure 9) would create defects in the dissolution of slag. These created defects could act as dislocations on which formation of etch pit can take place. Furthermore, the solution saturation is also reported to significantly affect the dissolution of slag [55]. Therefore, it is conceivable to use the dissolution theory-based mechanism to interpret the dissolution of slag.

**Figure 11:**
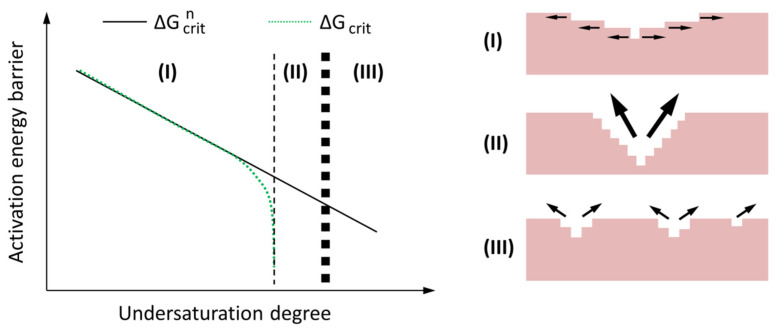
Schematic illustration of the two activation energy barriers ΔG_crit_^n^ (vacancy islands) and ΔG_crit_ (etch pit) as a function of the undersaturation degree. (after [54]).

Among the three dissolution forms, the first two are fast dissolution processes while the third is a slow dissolution process. The first two dissolution forms have activation energies, i.e., ΔG_crit_^n^ and ΔG_crit_, respectively. Prior to the onset of dissolution through the first two forms, the activation energy barriers must be removed. Figure 11 schematically demonstrates these two activation energy barriers as a function of the undersaturation of solution. A reduction of the undersaturation degree leads to an increase of the activation energy barrier. The undersaturation of the solution provides the energy to remove the activation energy barriers for dissolving through the formation of vacancy islands and etch pit at dislocations.

As shown in Figure 11, there are three regimes, i.e., regimes I, II and III, and their corresponding rate controlling mechanisms are step retreat, etch pit formation and vacancy islands formation, respectively. In regime III the undersaturation degree is very large (for example at the beginning of dissolution of slag), providing sufficient energy to remove the activation energy barrier for formation of vacancy islands on the smooth surface of slag. As the undersaturation moves from regime III to regime II, the energy provided by undersaturation decreases and is not able to remove the activation energy barrier of vacancy islands formation on the smooth surface of slag. On the other hand, etch pits is able to form at the dislocations or defects produced in regime III. When the undersaturation moves to regime I, the provided energy by undersaturation continues to decrease and it is not possible anymore for the formation of etch pits at dislocations or defects. In regime I the dissolution rate is low, since the dissolution is limited to the step retreat. It should be pointed out that the step retreat process is active during the whole dissolution process. In regimes II and III, however, the contribution of step retreat to the overall dissolution rate is small.

### 4.3. Interpreting the Action Mechanism of Soluble Si in the Dissolution of Slag in Alkaline Solution

**Figure 12:**
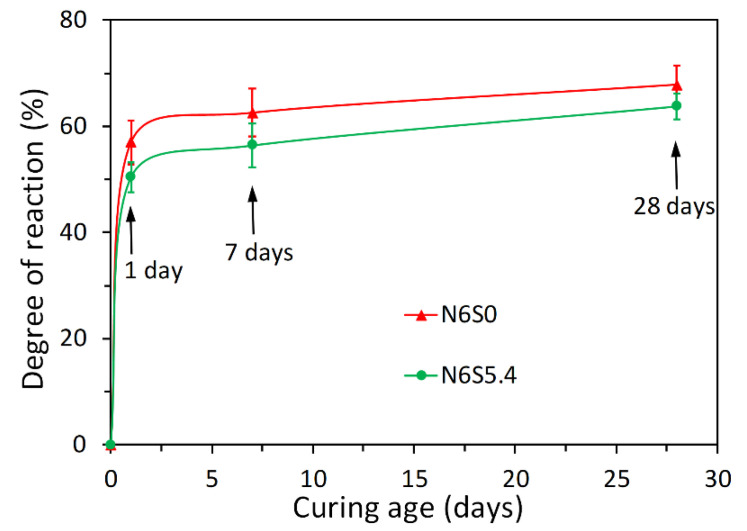
Degree of reaction of slag, derived from SEM-image analysis, for N6S0 and N6S5.4.

When slag was brought into contact with the NaOH solution with soluble Si, the soluble Si led to a very low undersaturation degree with respect to Si. As a result, the undersaturation was unlikely to supply sufficient energy to remove the activation energy barriers and thus it is difficult for vacancy islands and etch pits to form spontaneously on the leached surface layer. Then the dissolution moves rapidly to regime I, in which the dissolution is limited to step retreat. Therefore, the dissolution was slow and thus the dissolution of slag was retarded. Consequently, the concentrations of Ca and Al were smaller in the solution with soluble Si than those without soluble Si. The retarding effect of soluble Si on the reaction of slag can be further confirmed by the degree of reaction of slag as shown in Figure 12. It can be seen that the degree of reaction of slag was always smaller for sodium silicate activated slag than that for sodium hydroxide activated slag with the same content of Na_2_O.

### 4.4. Interpreting the Effects of Soluble Ca and Al in Solution on the Reaction of Slag

**Figure 13:**
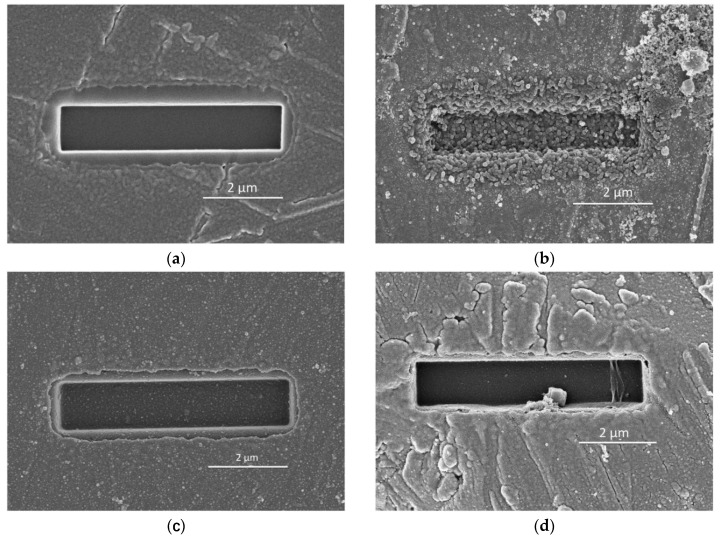
Micro-reactors: (**a**) gaps before immersion in solution, (**b**) after 2 days in 0.1 M KOH solution, (**c**) after 2 days in 0.1 M KOH + 20 mM CaCl_2_ solution and (**d**) after 2 days in 0.1 M KOH + 60 mM NaAlO_2_ solution. (cited from [55]).

Suraneni et al. used a micro-reactor approach to study the reactions of slag in alkaline solutions with soluble Ca and Al [55]. Figure 13 shows the micro-reactors before immersion in solution (Figure 13a), after 2 days in 0.1 M KOH solution (Figure 13b), after 2 days in 0.1 M KOH + 20 mM CaCl_2_ solution (Figure 13c) and after 2 days in 0.1 M KOH + 60 NaAlO_2_ solution (Figure 13d). Before immersion in solutions, the base and walls of the gaps were completely smooth. After 2 days of immersion in 0.1 M KOH solution, the gap showed clear growth of reaction products (globules). After 2 days of immersion in 0.1 M KOH solution with soluble Ca and Al, by contrast, the degree of dissolution of slag was significantly reduced, the walls were smooth and there was not much reaction products formed in the gap. It can be clearly seen that the soluble Ca and Al in solution significantly slowed down the reaction of slag.

The inhibiting effect of soluble Ca and Al on the reaction of slag may be also interpreted by the dissolution theory-based mechanism. The soluble Ca and Al in solution decreased the undersaturation with respect to the anhydrous phases. This made it difficult to provide sufficient activation energy to overcome the energy barriers for vacancy island formation and etch pit formation at dislocations. The dissolution of slag was mainly limited to the step retreat with very low dissolution rate. Consequently, the reaction of slag was slowed down. It should be noted that the formation of covalent bonds between adsorbed Al species and hydroxylated silicate surfaces, which was found in the dissolution of tricalcium silicate [56], might also act in the dissolution of slag. This action can lead to the inhibiting effect of soluble Al on the reaction of slag.

### 4.5. Interpreting the Reaction Process and Origin of the Induction Period of Alkali-Activated Slag

The good interpretation of the action mechanisms of soluble Si, Ca and Al in the dissolution or reaction of slag using the three forms of dissolution demonstrates that this dissolution theory based mechanism is applicable for describing the reaction process of alkali-activated slag.

In the beginning of NaOH activated slag paste, undersaturation degree was very large since there were no soluble Si, Al, Ca and Mg in the activating solution. The undersaturation was able to provide sufficient energy to remove activation energy barriers and thus all the three dissolution forms took place. This resulted in rapid dissolution of constituents in slag, which was accompanied with rapid release of heat (reflected by P_1_, see Figure 3a). The elements released into solution then existed as soluble elements (normally as ions). These soluble elements then reduced the undersaturation degree. As a result, the dissolution gradually moved from regime III to regime II, in which the dissolution via vacancy islands formation was not likely to take place anymore. In the meanwhile, the preferential dissolution of modifying elements and Al led to an increase of the thickness of the leached surface layer. The leached surface layer became stable when the dissolution of modifying elements and Al was in a dynamic equilibrium with the dissolution of Si. When the concentrations of soluble elements in solution increased towards saturation or oversaturation, reaction products started to precipitate. The precipitation of reaction products consumed soluble elements in solution, which increased the undersaturation degree and thus accelerated the dissolution of slag. The dissolution of slag then led to an increase of the concentrations of soluble elements in solution, facilitating the precipitation of reaction products. In this way the dissolution of slag and formation of reaction products progressed interdependently through the soluble elements in solution. Thus, interdependent processes led to a rapid growth of reaction products, which can be reflected by P_2_ in Figure 3a.

**Figure 14:**
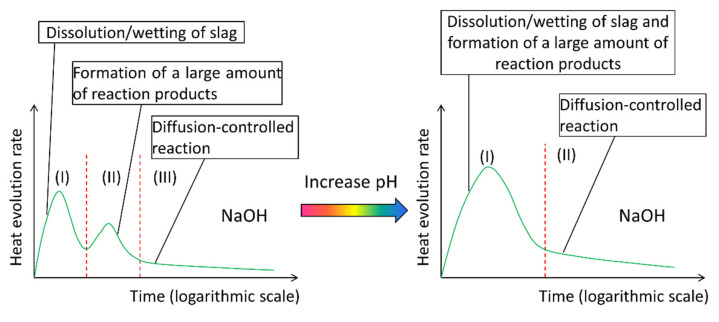
Schematic illustration of the effect of increasing the pH of NaOH activator on the reaction kinetics of NaOH activated slag paste.

Since these two interdependent processes facilitated each other, P_2_ occurred closely after P_1_. As a result, the induction period did not appear between P_1_ and P_2_ in the sodium hydroxide activated slag paste. It should be noted that a higher alkalinity or Na_2_O content of sodium hydroxide activator would accelerate these two interdependent processes. This can be confirmed by the observations in Figure 3a, i.e., the magnitude of P_2_ became larger and P_2_ also appeared earlier when the Na_2_O content increased. As schematically shown in Figure 14, it can be inferred that P_2_ will rise and advance earlier to merge with P_1_ when the alkalinity continues to increase. This inference has been evidenced by the isothermal calorimetric results in [21].

**Figure 15:**
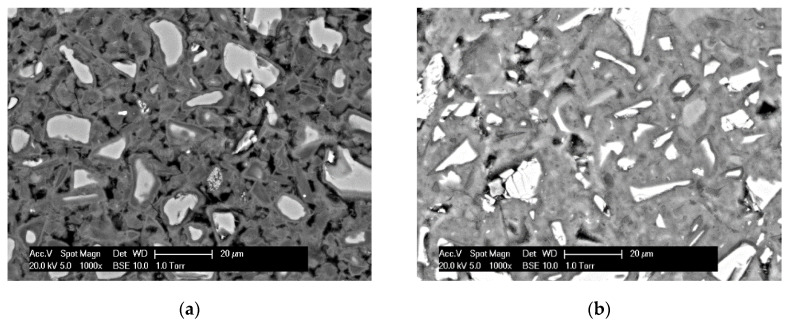
Micrographs, derived by SEM, for N4S0 (**a**) and N4S5.4 (**b**) at 28 days. N and S indicate weight percentages of Na_2_O and SiO_2_ with respect to slag, respectively.

In sodium hydroxide activated slag paste, only slag grains acted as nucleation sites. This led to the formation of reaction products mainly on the surface of slag grains. The continuous formation of reaction products around slag resulted in the layers of reaction products, as shown in Figure 15a. This is in line with the SEM observations in [32,45,46]. The layers of reaction products then acted as diffusion barriers for the diffusion of OH^−^ from the solution to the undissolved slag. Consequently, the dissolution of slag slowed down, which hence retarded the chemical reactions among soluble elements. Accompanying the decelerated dissolution and chemical reactions, heat evolution release also slowed down after P_2_. Then the reaction of slag gradually moved into a steady period in which the dissolution and reaction of slag was controlled by a diffusion process of hydroxide ions through the layers of reaction products.

In sodium silicate activated slag paste, undersaturation degrees of the modifying elements (Ca, Mg, and K) and Al were initially very large in the beginning. The undersaturation was able to provide sufficient energy to remove activation energy barriers and thus all the three dissolution forms took place for the dissolution of these elements. This resulted in rapid release of the modifying elements and Al, which was accompanied with rapid release of heat (reflected by P_1_, see Figure 3b). However, the soluble Si in sodium silicate activator led to a very low undersaturation degree with respect to Si. As a result, the undersaturation with respect to Si could not supply sufficient energy to remove the activation energy barriers for the dissolution of Si via formation of vacancy islands and etch pits on the leached surface layer. The dissolution of Si on the leached surface layer occurred in regime I. In regime I, the leached surface layer dissolved at a low rate since the dissolution was limited to the step retreat. Therefore, the modifying elements and Al dissolved faster than Si in the leached surface layer. This led to a continuous growth of the leached surface layer, which decreased the diffusion rate of modifying elements. This, therefore, slowed down the release of modifying elements. As a result, the reaction of slag came into an induction period.

In the meanwhile, the slowly released Ca and Al during the induction period gradually reacted with soluble Si to produce reaction products, which progressively consumed the soluble elements, in particular of the soluble Si. The consumption of soluble elements increased the undersaturation. When the undersaturation with respect to Si increased to provide sufficient energy, the activation energy barrier could be removed and the dissolution of Si could take place via formation of etch pits on the leached surface layer. This decreased the thickness of the leached surface layer, which then increased the diffusion rate of modifying elements through the leached surface layer. As a result, the dissolution of unreacted slag was accelerated. The dissolution of the leached surface layer and unreacted slag then released modifying elements, Al and Si into solution. This increased the concentrations of modifying elements, Al and Si towards saturation or oversaturation. Then reaction products grew rapidly. The fast growth of reaction products reflected intensive reactions, as characterized by P_2_ in Figure 3b.

In addition to slag grains the soluble Si in solution could also act as nucleation sites [57,58]. This led to formation of reaction products not only around slag grains but also in the solution space [53,54] (as seen in Figure 15b). With the continuous formation of reaction products, the microstructure of sodium silicate activated paste became denser. As a result, more and more diffusion paths of OH^−^ from solution to unreacted slag were blocked. Then the dissolution of slag and reactions of soluble elements moved into the steady period controlled by a diffusion process. Therefore, the heat evolution rate decreased gradually after P_2_.

## 5. Conclusions

This study used isothermal calorimetry to monitor the reaction kinetics of alkali-activated slag pastes and carried out a dissolution test to investigate the role of soluble Si in the dissolution of slag in alkaline solution. The origin of the induction period and action mechanism of soluble Si on the dissolution of slag were interpreted explicitly for the first time in this study. The main conclusions can be drawn as follows:

1. The heat release rate curves of alkali-activated slag paste depended on the presence of soluble Si in alkaline activator. For NaOH activated slag paste, three reaction periods were identified according to P_1_ and P_2_. For sodium silicate activated slag paste, one more reaction period was found between P_1_ and P_2_, i.e., the induction period.
2. The dissolution test revealed that the soluble Si in alkaline solution slowed down the dissolution of slag. The action mechanism of soluble Si, Ca and Al in the dissolution or reaction of slag was well interpreted by the dissolution theory-based mechanism. This demonstrates that the dissolution theory-based mechanism is applicable for describing the reaction process of alkali-activated slag paste, particularly for understanding the induction period.
3. In NaOH activated slag paste, a large undersaturation degree resulted in rapid dissolution of slag, leading to no noticeable induction period. In sodium silicate activated slag paste, the undersaturation with respect to Si was very low that it could not supply sufficient energy to remove the activation energy barriers for the formation of vacancy islands and etch pits on the surface of slag. This retarded the dissolution of slag, as a result of which, an induction period occurred.

## Figures and Tables

**Table 1:** Chemical composition of GGBFS.

Oxide	SiO_2_	CaO	Al_2_O_3_	MgO	Fe_2_O_3_	SO_3_	K_2_O	TiO_2_	LOI *
Weight (%)	32.91	40.96	11.85	9.23	0.46	1.61	0.33	1.00	1.15

* LOI refers to loss on ignition.

**Table 2:** Mix compositions of alkali-activated slag pastes.

Mixture	Slag (g)	Na_2_O (g)	SiO_2_ (g)	*M_s_* ^†^	Water (g)
N4S0	100	4	0	0	40
N6S0	100	6	0	0	40
N8S0	100	8	0	0	40
N4S5.4	100	4	5.4	1.395	40
N6S5.4	100	6	5.4	0.93	40
N8S5.4	100	8	5.4	0.6975	40

^†^*M_s_* is the molar modulus of alkaline activator, calculated as: *M_s_* = SiO_2_ (mol)/Na_2_O (mol).

**Table 3:** Chemical reactions and equilibrium solubility products at 25 °C and 1 bar for eight CNASH_ss end-members and secondary reaction products in alkali-activated slag.

End-Member	Chemical Reactions	*Log K_s0_*
*CNASH gel ideal solid solution eight end-members, ‘CNASH_ss’ model* [36]		
5CA	(CaO)_1.25_·(Al_2_O_3_)_0.125_·(SiO_2_)·(H_2_O)_1.625_ $\Leftrightarrow \;1.25{\mathrm{Ca}}^{2+}+{\mathrm{SiO}}_{3}^{2-}+0.25{\mathrm{AlO}}_{2}^{-}+\;0.25{\mathrm{OH}}^{-}+1.5H_{2}O$	−10.75
INFCA	(CaO)·(Al_2_O_3_)_0.15625_·(SiO_2_)_1.1875_·(H_2_O)_1.65625_ $+0.6875{\mathrm{OH}}^{-}$ $\Leftrightarrow {\;\mathrm{Ca}}^{2+}+1.1875{\mathrm{SiO}}_{3}^{2-}+0.3125{\mathrm{AlO}}_{2}^{-}+2H_{2}O$	−8.90
5CNA	(CaO)_1.25_·(Na_2_O)_0.25_·(Al_2_O_3_)_0.125_·(SiO_2_)·(H_2_O)_1.25_ $\Leftrightarrow \;1.25{\mathrm{Ca}}^{2+}+{\mathrm{SiO}}_{3}^{2-}+0.25{\mathrm{AlO}}_{2}^{-}+0.5{\mathrm{Na}}^{+}+0.75{\mathrm{OH}}^{-}+H_{2}O$	−10.40
INFCNA	(CaO)·(Na_2_O)_0.34375_·(Al_2_O_3_)_0.15625_·(SiO_2_)_1.1875_·(H_2_O)_1.3_ $\Leftrightarrow {\;\mathrm{Ca}}^{2+}+1.1875{\mathrm{SiO}}_{3}^{2-}+0.3125{\mathrm{AlO}}_{2}^{-}+0.6875{\mathrm{Na}}^{+}+1.3125H_{2}O$	−10.00
INFCN	(CaO)·(Na_2_O)_0.3125_·(SiO_2_)_1.5_·(H_2_O)_1.1875_ $+\;0.375{\mathrm{OH}}^{-}$ $\Leftrightarrow {\;\mathrm{Ca}}^{2+}+1.5{\mathrm{SiO}}_{3}^{2-}+0.625{\mathrm{Na}}^{+}+1.375H_{2}O$	−10.70
T2C*	(CaO)_1.5_·(SiO_2_)·(H_2_O)_2.5_ $\Leftrightarrow \;1.5{\mathrm{Ca}}^{2+}+{\mathrm{SiO}}_{3}^{2-}+{\mathrm{OH}}^{-}+2H_{2}O$	−11.60
T5C*	(CaO)_1.25_·(SiO_2_)_1.25_·(H_2_O)_2._ $\Leftrightarrow \;1.25{\mathrm{Ca}}^{2+}+1.25{\mathrm{SiO}}_{3}^{2-}+2.5H_{2}O$	−10.50
TobH*	(CaO)·(SiO_2_)_1.5_·(H_2_O)_2.5_ $+{\mathrm{OH}}^{-}$ $\Leftrightarrow {\;\mathrm{Ca}}^{2+}+1.5{\mathrm{SiO}}_{3}^{2-}+3H_{2}O$	−7.90
*Crystalline reaction products in alkali-activated slag* [33,39]		
C_2_ASH_8_	(CaO)_2_·(Al_2_O_3_)·(SiO_2_)·(H_2_O)_8_ $\Leftrightarrow \;2{\mathrm{Ca}}^{2+}+2{\mathrm{AlO}}_{2}^{-}+{\mathrm{SiO}}_{3}^{2-}+8H_{2}O$	−19.10
C_3_AH_6_	(CaO)_3_·(Al_2_O_3_)·(H_2_O)_6_ $\Leftrightarrow \;3{\mathrm{Ca}}^{2+}+2{\mathrm{AlO}}_{2}^{-}+4{\mathrm{OH}}^{-}+4H_{2}O$	−20.85
Ca(OH)_2_	Ca(OH)_2_ $\Leftrightarrow {\;\mathrm{Ca}}^{2+}+2{\mathrm{OH}}^{-}$	−5.20
C_4_AH_13_	(CaO)_4_·(Al_2_O_3_)·(H_2_O)_13_ $\Leftrightarrow \;4{\mathrm{Ca}}^{2+}+2{\mathrm{AlO}}_{2}^{-}+6{\mathrm{OH}}^{-}+10H_{2}O$	−25.41

Note: 5CA and INFCA are two C-A-S-H end-members; 5CNA and INFCNA are two C-N-A-S-H end-members; INFCN a C-N-S-H end-member; T2C*, T5C* and TobH* are three C-S-H end-members [36].

**Table 4:** Measured elemental concentrations of Si, Al and Ca (mmol/L).

[Si]	Element	10 min	25 min	40 min	60 min	90 min	120 min
0 mmol/L	(Si)	0.0786	0.0961	0.1400	0.1929	0.2464	0.2857
	(Al)	0.0415	0.0459	0.0681	0.0900	0.1111	0.1270
	(Ca)	0.1300	0.1650	0.1775	0.1975	0.2500	0.2700
[Si]	element	10 min	30 min	60 min	90 min	120 min	
1 mmol/L	(Si)	1.4500	1.4821	1.5000	1.5429	1.5679	
	(Al)	0.0333	0.0296	0.0519	0.0556	0.0704	
	(Ca)	0.1400	0.1175	0.1350	0.1475	0.1625	
5 mmol/L	(Si)	5.1071	5.3214	5.0714	4.2143	4.7857	
	(Al)	0.0444	0.0407	0.0407	0.0593	0.0481	
	(Ca)	0.0575	0.1050	0.0850	0.0725	0.0925	
